# Place Matters: Geographic Distribution of Abortion Fund Services

**DOI:** 10.1177/24731242251369612

**Published:** 2025-08-22

**Authors:** Erin R. Johnson, Monica R. McLemore

**Affiliations:** ^1^ Department of Sociology, College of Liberal Arts, University of Southern Indiana, Evansville, Indiana, USA.; ^2^ Rory Meyers College of Nursing, New York, New York, USA.

**Keywords:** abortion, reproductive health, health care access, community-based organizations

## Abstract

**Background:**

Where you live impacts your access to all forms of health care, but abortion in particular. In response to restrictions on abortion, communities have organized to support those seeking abortion care via abortion funds. This study documents the services provided by these organizations and examines how they have been shaped by local conditions.

**Methods:**

Data come from a content analysis of the websites of all abortion funds affiliated with the National Network of Abortion Funds and interviews with 22 abortion fund leaders. Content analysis data presented include data about abortion funds’ catchment area and what services funds provide.

**Results:**

Types of support offered by abortion funds include procedure funding, practical support, emotional support, other sexual health services, and parenting support. Interviews with fund leaders show that these services are shaped by local policies, cultural and geographic specifics of their catchment area, local clinics, and ties to other organizations in funds’ communities.

**Discussion:**

Abortion funds are a diverse group of organizations that provide a variety of services to help patients overcome barriers to abortion. The geographic patterning of their services suggest that funds are responding to local environments, with the greatest variety of services being offered by funds in the South.

**Health Equity Implications:**

Abortion funds’ service offerings clearly respond to the barriers to abortion described in the literature, driven by the needs of their local communities. This is particularly relevant in a post-Dobbs environment, as patients in some parts of the country find abortion even farther out of their reach.

Where you live impacts your access to all forms of health care, but abortion in particular. In the US, abortion policy varies considerably at the state level, making abortion harder to access in some states than others. State laws requiring waiting periods or pre-abortion counseling make access logistically difficult, while Targeted Regulation of Abortion Provider (TRAP) laws (which impose burdensome standards on facilities) often force clinics to close or relocate.^1,2^ After the *Dobbs* decision^^a^^, these disparities became more stark as clusters of states severely limited abortion access.^3^

State policies also affect abortion patients’ ability to pay for care. The Hyde Amendment prevents federal money from funding abortion care except in rare circumstances.^4^ While 17 states require Medicaid to pay for medically necessary abortions and eight allow Medicaid to cover abortion care under limited circumstances, patients and providers struggle to access these options, putting abortion out of reach for many low-income people.^5–8^

In response to restrictions on abortion, communities have organized to support those seeking abortion care. Abortion funds are largely grassroots organizations that exist to help patients overcome barriers to abortion care through a variety of services. Since 1993, abortion funds have organized under the umbrella organization the National Network of Abortion Funds (NNAF). Abortion funds especially impact individuals from minoritized communities and those who face significant hardships in accessing care.^9–12^

Despite their importance within the abortion access ecosystem, few studies have centered on abortion funds. Those that have focus on a single fund or geographic group of funds. Few studies describe abortion funds as a category of organizations. This study uses data from a content analysis of fund websites and interviews with fund leaders to examine how abortion funds are rooted in the needs of communities by examining the geographic patterning of fund services and by using interview data to give context to these results.

## Methods

Analyzed data derive from two sources—content analysis of the websites of all NNAF-affiliated funds and interviews with 22 fund leaders. The content analysis sample was restricted to operating funds with English-language websites. Of the 100 funds listed on the NNAF website at the time of data collection, three were closed, one had no website, and one had only a Spanish-language website, leaving a sample size of 95 funds. The first author reviewed each website between April and July 2023, collecting data via a structured, mixed-methods survey instrument in Qualtrics.^13^

Content analysis data presented here include data about abortion funds’ catchment area and what services funds provide. Data about funds’ services were originally captured as website text and quantified via a sorting exercise. The first author read through the data, inductively developed a preliminary list of categories, and then sorted the data into these categories, setting aside data that fit into multiple categories or did not fit into any category. Once preliminary sorting was complete, the first author examined data that did not fit the initial set of categories and developed a revised set. The first author repeated this process until each datum fit into exactly one category. Quantitative data were cleaned in Excel and analyzed in Stata.

Interviews with fund leaders took place between November 1, 2023, and February 15, 2024. A “leader” was defined as anyone with significant decision-making authority, and participants could determine their own eligibility. Participants were recruited for 1-hour interviews via social media and snowball sampling. The authors chose this method due to concerns about gaining access to this busy and somewhat hard-to-reach population. Participants received a $50 gift card for time and effort. Ethics approval came from the Institutional Review Board at the University of California, San Francisco. Participants received a consent form to review in advance of interviews, and the interviewer obtained verbal consent before beginning the interview.

Interviews were recorded on Zoom and transcribed using the GoTranscript human transcription service.^14^ The first author developed the initial interview guide draft based on results from the content analysis and review of the literature. This was then iteratively edited by the authors. The interview guide covered personal history and motivations for fund work, fund practices, the leader’s experiences of *Dobbs*, and larger-picture questions about the funds’ long-term goals and social movement involvement. Interview data were coded and analyzed in MaxQDA using constructivist grounded theory methods. The authors met regularly to discuss coding progress and emerging findings.

The 22 interviewees represent 20 funds from all four regions of the US (as defined by the US Census Bureau): the Northeast (*n* = 10), the Midwest (*n* = 3), the South (*n* = 6), and the West (*n* = 2). One participant represented a fund with national reach. Participants’ funds work in states where abortion was legal up to or beyond the point of viability (*n* = 11), restricted between 6 weeks and 18 weeks (*n* = 3), and banned completely (*n* = 6), and one participant represented a regional fund working in states with a mix of policies. Six participants identified as Black, Hispanic, or mixed race, and 16 identified as white. One participant identified as a man; the rest identified as women. They held a variety of positions, including Board Officer (e.g., President; *n* = 6) or Board Member (*n* = 3); Director, Executive Director, or Deputy Director (*n* = 5); and Program Director, Manager, or Coordinator (*n* = 8). Participants are referred to by pseudonyms below, and potentially identifying details were removed from quotes.

## Results

### Abortion fund service types by US region

The data show most abortion funds operate within a specific geographic area—serving individuals within a city, county, state, or region. 49 of 50 states have at least one such fund. In addition, there are five national funds that serve people across the US who meet specific criteria (e.g., getting a telemedicine abortion). There are four international funds, which primarily serve patients outside of the US (See Table 1).

**Table 1.** Geographic Distribution of Abortion Funds and the Services They Provide (*N* = 95)

	Total (*N*)^a^	Procedure funding Row% (*N*)	Practical support Row% (*N*)	Emotional support Row% (*N*)	Sexual health services Row% (*N*)	Parenting support Row% (*N*)
Northeast	16	88% (14)	44% (7)	13% (2)	25% (4)	13% (2)
New England	11	82% (9)	36% (4)	18% (2)	36% (4)	18% (2)
Mid Atlantic	5	100% (5)	60% (3)	0% (0)	0% (0)	0% (0)
Midwest	25	92% (23)	56% (14)	8% (2)	28% (7)	8% (2)
East North Central	13	92% (12)	62% (8)	15% (2)	31% (4)	15% (2)
West North Central	16	88% (14)	50% (8)	0% (0)	25% (4)	0% (0)
South	34	85% (29)	62% (21)	24% (8)	35% (12)	9% (3)
South Atlantic	16	100% (16)	56% (9)	19% (3)	44% (4)	0% (0)
East South Central	9	78% (7)	67% (6)	33% (3)	25% (3)	33% (3)
West South Central	12	75% (9)	67% (8)	25% (3)	20% (4)	0% (0)
West	20	95% (19)	65% (13)	25% (5)	19% (3)	0% (0)
Mountain	16	100% (16)	56% (9)	19% (3)	20% (1)	0% (0)
Pacific	5	80% (4)	100% (5)	60% (3)	33% (1)	0% (0)
National	5	80% (4)	60% (3)	20% (1)	20% (1)	20% (1)
International	3	100% (3)	33% (1)	0% (0)	0% (0)	0% (0)
Total	95	91% (86)	59% (56)	18% (17)	28% (27)	6% (6)

^a^ Since some funds serve states across multiple regions or subregions and one fund serves cities on either side of the US-Mexico border, columns do not tally to the total at the bottom.

Most abortion funds (91%) fulfill their eponymous purpose—helping people pay for abortion procedures. Specifically, all funds in the Mid Atlantic, South Atlantic, and Mountain divisions provide this service, as do all international funds for which data were available. In contrast, approximately a quarter of funds in the remaining Southern divisions do not fund abortions at all. However, just as cost is not the only barrier to abortion care, organizations that self-identify as abortion funds do not only fund abortions. These organizations also provide practical (56%) and emotional (18%) support in accessing abortion care. Some funds also assist with other types of sexual and reproductive health needs (28%) or support individuals who choose to parent (6%). Approximately two-thirds of NNAF affiliatedfunds (63 of 95 funds with data) provide services in addition to or instead of funding abortion procedures.

Practical support is the second most common service provided and includes assistance with long-distance travel (34% of abortion funds), rides to and from clinics (33%), lodging (33%), meals (23%), childcare (20%), miscellaneous expenses (18%), and informational assistance via patient navigation and referrals (28%). Table 2 provides a regional breakdown of services. Notably, funds in the Northeastern US less frequently provide these services, with no Northeastern funds providing meals, childcare, or miscellaneous expenses, and one quarter or fewer of funds from that region providing any type of practical support.

**Table 2.** Geographic Distribution of Practical Support Services (*N* = 95)

	Travel Row% (*N*)	Rides Row% (*N*)	Lodging Row% (*N*)	Meals Row% (*N*)	Child care Row% (*N*)	Other costs Row% (*N*)	Navigation and referrals Row% (*N*)
Northeast (*N* = 16)	6% (1)	19% (3)	13% (2)	0% (0)	0% (0)	0% (0)	25% (4)
New England (*N* = 11)	0% (0)	18% (2)	18% (2)	0% (0)	0% (0)	0% (0)	27% (3)
Mid Atlantic (*N* = 5)	20% (1)	20% (1)	0% (0)	0% (0)	0% (0)	0% (0)	20% (1)
Midwest (*N* = 25)	36% (9)	28% (7)	32% (8)	16% (4)	20% (5)	20% (5)	28% (7)
East North Central (*N* = 13)	38% (5)	23% (3)	23% (3)	8% (1)	15% (2)	15% (2)	31% (4)
West North Central (*N* = 16)	38% (6)	25% (4)	38% (6)	25% (4)	25% (4)	31% (5)	31% (5)
South (*N* = 34)	30% (10)	35% (12)	29% (10)	21% (7)	21% (7)	21% (7)	24% (8)
South Atlantic (*N* = 16)	31% (5)	44% (7)	38% (6)	31% (5)	31% (5)	31% (5)	6% (1)
East South Central (*N* = 9)	25% (2)	33% (3)	22% (2)	11% (1)	22% (2)	22% (2)	44% (4)
West South Central (*N* = 12)	33% (4)	33% (4)	33% (4)	17% (2)	8% (1)	8% (1)	33% (3)
West (*N* = 20)	50% (10)	50% (10)	50% (10)	45% (9)	25% (5)	25% (5)	30% (6)
Mountain (*N* = 16)	38% (6)	38% (6)	38% (6)	31% (5)	13% (2)	19% (3)	25% (4)
Pacific (*N* = 5)	100% (5)	100% (5)	100% (5)	100% (5)	60% (3)	60% (3)	40% (2)
National (*N* = 4)	60% (3)	20% (1)	40% (2)	40% (2)	40% (2)	0% (0)	40% (2)
International (*N* = 3)	0% (0)	0% (0)	33% (1)	0% (0)	0% (0)	0% (0)	33% (1)
Total (*N* = 95)	34% (32)	33% (31)	33% (31)	23% (22)	20% (19)	18% (17)	28% (27)

The remaining service categories are less common, but their existence speaks to the diversity of work that funds do and the diversity of their communities. In response to the stigma that often accompanies accessing abortion care, funds provide emotional support for people seeking care in a variety of ways. Emotional support services are more commonly offered by funds in the South and West (Table 3). In addition to helping individuals seeking abortion care, some funds provide help with other sexual and reproductive health services or provide support for individuals who choose to parent. Sexual health services are offered by a fairly similar percentage of funds across regions, although they are slightly more common in the South (Table 3). Parenting support services are the least common type of support offered, and no funds in the Western US offer these services (Table 3). Table 4 contains a breakdown of the types of support offered in each of these categories.

**Table 3.** Geographic Distribution of Emotional, Sexual Health, and Parenting Support Services (*N* = 95)

	Helpline Row% (*N*)	Abortion doula care Row% (*N*)	Other emotional support Row% (*N*)	Clinical contraceptive care Row% (*N*)	Gender affirming care Row% (*N*)	Emergency contraceptives or sexual health kits Row% (*N*)	Birth doula care Row% (*N*)	Infant supplies Row% (*N*)
Northeast (*N* = 16)	6% (1)	0% (0)	7% (1)	13% (2)	13% (2)	19% (3)	0% (0)	13% (2)
New England (*N* = 11)	9% (1)	0% (0)	10% (1)	18% (2)	18% (2)	27% (3)	0% (0)	18% (2)
Mid Atlantic (*N* = 5)	0% (0)	0% (0)	0% (0)	0% (0)	0% (0)	0% (0)	0% (0)	0% (0)
Midwest (*N* = 25)	8% (2)	0% (0)	0% (0)	12% (3)	4% (1)	24% (6)	4% (1)	4% (1)
East North Central (*N* = 13)	17% (2)	0% (0)	0% (0)	8% (1)	8% (1)	31% (4)	8% (1)	8% (1)
West North Central (*N* = 16)	0% (0)	0% (0)	0% (0)	13% (2)	0% (0)	19% (3)	0% (0)	0% (0)
South (*N* = 34)	0% (0)	8% (3)	16% (5)	18% (6)	6% (2)	26% (9)	3% (1)	9% (3)
South Atlantic (*N* = 16)	0% (0)	0% (0)	19% (3)	19% (3)	0% (0)	25% (4)	0% (0)	0% (0)
East South Central (*N* = 9)	0% (0)	22% (2)	14% (1)	11% (1)	11% (1)	44% (4)	11% (1)	33% (3)
West South Central (*N* = 12)	0% (0)	8% (1)	18% (2)	17% (2)	8% (1)	17% (2)	0% (0)	0% (0)
West (*N* = 20)	10% (2)	15% (3)	6% (1)	0% (0)	0% (0)	20% (4)	0% (0)	0% (0)
Mountain (*N* = 16)	6% (1)	13% (2)	0% (0)	0% (0)	0% (0)	19% (3)	0% (0)	0% (0)
Pacific (*N* = 5)	20% (1)	40% (2)	33% (1)	0% (0)	0% (0)	20% (1)	0% (0)	0% (0)
National (*N* = 4)	20% (1)	0% (0)	0% (0)	0% (0)	0% (0)	1% (20)	20% (1)	0% (0)
International (*N* = 3)	0% (0)	0% (0)	0% (0)	33% (1)	0% (0)	33% (1)	0% (0)	0% (0)
Total (*N* = 95)	6% (6)	6% (6)	7% (6)	12% (11)	4% (4)	23% (2)	3% (3)	4% (4)

**Table 4.** Detailed Descriptions of Types of Support Offered

Practical support	
Long-distance travel	Fund pays for travel by plane, train, or bus; volunteer drivers take patients to appointments in another city; fund provides money so patient can pay for gas
Rides	Fund provides money so patient can pay for gas or ride-sharing services; volunteer drivers take patients to appointments in the city where they live
Lodging	Fund pays for hotel; volunteers host patients in their homes
Meals	Fund provides cash or gift cards to pay for meals; volunteers host patients in their homes for meals
Child care	Fund provides cash or gift cards to pay for child care
Other costs	Fund covers additional clinic-based costs such as ultrasounds, Rhogam injections, or testing and treatment for STIs; fund covers incidental costs associated with travel or the supplies needed after an abortion such as menstrual products, over the counter pain medication, and heating pads
Navigation and referrals	Fund provides patient navigation, referrals to clinics or other funding sources, or legal support services
Emotional support	
Helpline	Funds train staff or volunteers to provide emotional support via a helpline
Abortion Doula Care	Funds provide paid or volunteer abortion doulas for clients
Other Support	Funds provide clinic escorts, peer support groups, or unspecified forms of emotional support
Sexual Health Support	
Clinical contraceptive care	Funds pay for a doctor’s visit and the cost of prescription contraceptives
Gender affirming care	Funds pay for gender affirming care such as hormone injections and surgeries
Emergency contraceptives or sexual health kits	Funds distribute emergency contraceptives (most typically Plan B) or sexual health kits (usually containing an emergency contraceptive, pregnancy tests, and condoms) via community partners such as other non-profits and local businesses or by mail
Parenting support	
Birth doula care	Funds provide paid or volunteer birth doulas for clients
Infant supplies	Funds provide infant supplies such as diapers and formula or nursing supplies such as breast pumps and nursing pads
Parenting support groups	Funds host parenting support groups

Funds may not always provide a comprehensive list of their services online. In two interviews, participants stated that their funds offered services not listed on their websites. When probed about this discrepancy, participants were uncertain about the cause. However, in other conversations, fund leaders expressed the value of flexibility, which allows funds to respond directly to the needs of people seeking abortion care. Chloe explained that a client’s needs may not relate directly to abortion access: “People don’t just need help with their abortions. We see people who are struggling with housing, and it’s all this other stuff on top of it … When we started, we made it so that we’re flexible. We didn’t want to put limits on what we’re able to help with.” (Midwest, Director, White)

### Fund leader discussions of the importance of place for abortion funds

While the analysis above demonstrates that services provided by abortion funds are influenced by geography, this quantitative data cannot explain emergent patterns. Interviews with abortion fund leaders make clear, however, that funds are deeply rooted in and responsive to local conditions. As Heather noted, “Arizona is different than Minnesota, which is different than Mississippi. We are the ones that know what our communities need … people … that have lived experience under whatever culture and legislature.” (National, Program Coordinator, White)

Funds are shaped by evolving local policies in both positive and negative ways. Heather—who previously worked at a statewide fund—recalled that when the implementation of TRAP laws in their state created increased costs for the local clinic, they saw a significant increase in requests for assistance, forcing the fund to increase its fundraising and strengthen its organization infrastructure to meet demand. (National, Program Coordinator, White) Other respondents recalled that their funds were able to pivot after the state implemented laws expanding Medicaid abortion coverage. Layla explained that the state’s Medicaid law allowed them to go from “only being able to fund second trimester abortions … [for] a certain amount of people to then … being able to support everyone who reaches out.” (Midwest, Deputy Director, Black)

Funds are also shaped by the cultural and geographic peculiarities of their catchment area. Leanne explained that her fund had chosen to provide birth control due to local conditions that made reproductive health care hard to access in clients’ communities. “Somebody might not feel comfortable picking up their birth control prescription from their local pharmacist when that pharmacist is also sitting in the church behind them and gets brunch with their mom.” However, the weather in her rural state often made it hard for people to get to clinics in other towns. Because of this, the fund wanted to help clients get everything they needed while they were there for an abortion. Leanne noted that an abortion appointment was also a time when clients were more open to trying birth control. “When folks face so many barriers to having an abortion, they’re more apt to pursue a birth control method. That’s a big wrap-around, but that’s where we’re at.” (Midwest, President, White)

In addition to the local environment, abortion funds are influenced by other organizations in their communities, particularly local clinics. Chloe described how her understanding of the clinic landscape allowed her to help redirect people with a later gestational age to the best location to receive care, saying, “When people called me from [city] … looking to schedule an appointment at the [local] clinic, I’m like, ‘No, please go to [neighboring state], because you might have to drive an extra hour, but you only have to go one time. If you are past 13 weeks and 6 days, they will still be able to see you.’” (Midwest, Director, White) Amanda explained how her fund had increased the number of callers by building relationships with clinics and making sure staff knew help was available. Now, she says, “oftentimes folks already have appointments when they’re calling us,” and their call volume had increased by fivefold, even before the *Dobbs* decision. (West, Executive Director, Mixed Race) Having a close relationship with clinics also means that those clinics can reach out when they have patients who may need extra assistance. Heather recalled that when she was managing her statewide fund’s warmline, “I also would get direct text requests from the docs at [clinic] … when things happened … People who were incarcerated, people who had bedbugs and could not be seen until that got taken care of.” (National, Program Coordinator, White)

Working closely with local clinics also ensures the fund is acting as a responsible steward of its resources by protecting against fraud and using each dollar for maximum impact. Caitlin recalled when their caller volume began to outstrip their ability to offer services. One way the fund continued to help more callers was to make sure people were being seen at clinics that accepted their insurance—including Medicaid. Although she admitted that the additional steps took a toll on both callers and volunteers, she explained, “We’re saving money with these new policies,” which allows them to help more people. (Northeast, Executive Director, Mixed Race) Chloe described how knowing the local clinic landscape allowed them to catch scammers as abortion funds gained more attention after the *Dobbs* decision. “They would be like, ‘Oh, well, I have an appointment in [city] on this day’ … I can go and see that [clinic] isn’t even open on that day … I have that backdoor knowledge, but if you don’t know that about clinics … then it’s easier to get by with that kind of stuff.” (Midwest, Director, White)

In addition to working with clinics, funds develop close ties with their broader communities. Many funds table at local events—including Pride celebrations—or partner with college student organizations to offer free Plan B and condoms and raise awareness about their other services. Several funds work with local transgender support organizations or health clinics to ensure that trans, nonbinary, and intersex people seeking abortion care receive respectful care. Whitney explained that her community outreach work for her fund had allowed her to bring resources to her historically Black neighborhood through conversations at community events and local barbershops: “Having those conversations with my community, it’s been fucking great. I love it … Being the abortion lady has just been fun.” (South, Program Coordinator, Black)

Because they are grounded in the local context, funds provide a sense of community support and approval, allowing them to destigmatize the abortion decision. Participants explained the importance of having local people work the fund hotlines. Maya elaborated, “there’s a lot of nuance based on state. Just going from [city in her state] to [other city in the state], you’re already introduced to three different cultures. That cultural aspect of understanding is how we’re engaging with our callers.” (West, Board Member, Black) Heather explained that this cultural common ground is particularly important when they send clients out of state, saying, “We built our program around knowing that even to the point that voice call with somebody who sounds like home is part of the support that we offer people, even if they’re not getting funding from us.” (National, Program Coordinator, White) Participants linked this community care aspect of the funds’ work to destigmatization, with Leanne saying, “I think that abortion funds are the best representation of community support we have, period … Abortion funds don’t give a fuck. They’re so in your face about it … I think that abortion funds will be credited to the destigmatization of abortion and the normalizing of abortion.” (Midwest, President, White)

## Discussion

This data show that abortion funds exist throughout the US and in other parts of the world, but there is no single fund model. Instead, abortion funds are a diverse group of organizations that provide a variety of services to help patients overcome barriers to accessing abortion with patterned variability in their geographic distribution, suggesting that funds are affected by regional and sub-regional environments. This makes sense in the context of abortion policy and the abortion access landscape, which varies considerably across the US.

Funds in the South are particularly innovative in their offerings. More funds in the South focus exclusively on services other than procedural funding compared to other regions. The South also offers sexual health services more frequently than any other regions and emotional support services more frequently than any other region but one. This is consistent with the South’s anti-abortion policy innovations. In addition, Southern states tend to be large and rural, which makes the practical barriers to accessing abortion care equal to the financial barriers. These challenges have driven the formation of local communities of care, which have collaborated to develop creative solutions to the problem of accessing abortion in this hostile region.

Fund leaders give context to these quantitative data by explaining how local conditions shape abortion funds. This grounding in the local context allows funds to meet the needs of individuals seeking abortion care in their community. In addition, because funds are grounded in the local context, they allow people receiving assistance to feel accepted by their communities as they are making the choice to terminate a pregnancy—decreasing potential stigma.

### Limitations

These data are a point-in-time snapshot of publicly available information. As noted above, abortion fund websites may not perfectly reflect the services they offer. Additionally, funds may have changed their service offerings, opened, closed, or changed their affiliation with NNAF since data collection. Clients may choose not to use cash provided by these organizations as intended, which is not reflected in this analysis. Finally, this analysis excludes data from funds not affiliated with NNAF at the time of data collection.

## Health Equity Implications

These data show that abortion funds have been directly shaped by the needs of their community. Abortion funds’ service offerings clearly respond to the barriers to abortion described in the literature—high cost of care, need for assistance planning and travel costs, and need for patient navigation and emotional support.^3,15–17^ However, funds also emphasize the value of flexibility in their offerings in order to meet patient needs. Beyond supporting abortion access, abortion funds provide support for fertility management, sexual health, and even parenting, allowing them to provide wrap-around care for individuals seeking abortion services or to address a broader range of needs within their communities. This is particularly relevant in the wake of the Dobbs decision, as funds in states with complete abortion bans have had to pivot to adjust to new policy and clinic landscapes and may need new ways to reach their audience.

## Notes

^a^
The U.S. Supreme Court’s decision in *Dobbs v. Jackson Women’s Health Organization* overturned the precedent set in *Roe v. Wade* that abortion access until viability is a Constitutional right, allowing states and the federal government to make laws restricting abortion access before viability.

## Abbreviations Used

NNAF: National Network of Abortion Funds
TRAP: Targeted Regulation of Abortion Provider
US: United States
